# CMR fluoroscopy right heart catheterization for cardiac output and pulmonary vascular resistance: results in 102 patients

**DOI:** 10.1186/s12968-017-0366-2

**Published:** 2017-07-27

**Authors:** Toby Rogers, Kanishka Ratnayaka, Jaffar M. Khan, Annette Stine, William H. Schenke, Laurie P. Grant, Jonathan R. Mazal, Elena K. Grant, Adrienne Campbell-Washburn, Michael S. Hansen, Rajiv Ramasawmy, Daniel A. Herzka, Hui Xue, Peter Kellman, Anthony Z. Faranesh, Robert J. Lederman

**Affiliations:** 1 Cardiovascular and Pulmonary Branch, Division of Intramural Research, National Heart Lung and Blood Institute, National Institutes of Health, Bethesda, MD USA; 2 Department of Cardiology, Rady Children’s Hospital, San Diego, CA USA; 3 Department of Cardiology, Children’s National Medical Center, Washington, DC USA; 4 Division of Intramural Research, National Heart Lung and Blood Institute, National Institutes of Health, Building 10, Room 2c713, Bethesda, MD 20892-1538 USA

**Keywords:** Interventional MRI catheterization, Right heart catheterization, Invasive hemodynamics, Phase contrast MRI flow, Real-time MRI, CMR, Cardiac MRI

## Abstract

**Background:**

Quantification of cardiac output and pulmonary vascular resistance (PVR) are critical components of invasive hemodynamic assessment, and can be measured concurrently with pressures using phase contrast CMR flow during real-time CMR guided cardiac catheterization.

**Methods:**

One hundred two consecutive patients underwent CMR fluoroscopy guided right heart catheterization (RHC) with simultaneous measurement of pressure, cardiac output and pulmonary vascular resistance using CMR flow and the Fick principle for comparison. Procedural success, catheterization time and adverse events were prospectively collected.

**Results:**

RHC was successfully completed in 97/102 (95.1%) patients without complication. Catheterization time was 20 ± 11 min. In patients with and without pulmonary hypertension, baseline mean pulmonary artery pressure was 39 ± 12 mmHg vs. 18 ± 4 mmHg (*p* < 0.001), right ventricular (RV) end diastolic volume was 104 ± 64 vs. 74 ± 24 (*p* = 0.02), and RV end-systolic volume was 49 ± 30 vs. 31 ± 13 (*p* = 0.004) respectively. 103 paired cardiac output and 99 paired PVR calculations across multiple conditions were analyzed. At baseline, the bias between cardiac output by CMR and Fick was 5.9% with limits of agreement −38.3% and 50.2% with *r* = 0.81 (*p* < 0.001). The bias between PVR by CMR and Fick was −0.02 WU.m^2^ with limits of agreement −2.6 and 2.5 WU.m^2^ with *r* = 0.98 (*p* < 0.001). Correlation coefficients were lower and limits of agreement wider during physiological provocation with inhaled 100% oxygen and 40 ppm nitric oxide.

**Conclusions:**

CMR fluoroscopy guided cardiac catheterization is safe, with acceptable procedure times and high procedural success rate. Cardiac output and PVR measurements using CMR flow correlated well with the Fick at baseline and are likely more accurate during physiological provocation with supplemental high-concentration inhaled oxygen.

**Trial registration:**

Clinicaltrials.gov NCT01287026, registered January 25, 2011.

## Background

Quantification of cardiac output in the cardiac catheterization laboratory is a central component of hemodynamic assessment. The most commonly used methods, thermodilution and the Fick principle, are subject to substantial error from intrinsic inaccuracy or imprecise assumptions. Thermodilution is particularly unreliable in the setting of intracardiac shunts or tricuspid regurgitation [1]. Calculations using the Fick principle require knowledge of oxygen consumption (VO_2_) that is rarely measured directly, and instead is usually assumed from age, sex and heart rate [2–4]. Both methods commonly are inaccurate by over 25%.

In contrast, velocity encoded phase contrast cardiovascular magnetic resonance (CMR) is a well-validated technique to directly measure stroke volume through a major vessel [5], for example the pulmonary artery or aorta, from which cardiac output can be derived. CMR flow measurements have been shown to be superior to Fick for calculation of hemodynamic parameters such as cardiac output and pulmonary vascular resistance that establish diagnosis, guide treatment and inform prognosis [6].

Pressures and cardiac output should preferably be measured simultaneously. Right heart catheterization (RHC) can be safely performed in the CMR scanner using CMR fluoroscopy (also known as real-time CMR) to guide catheter navigation through the vasculature and cardiac chambers [7, 8], avoiding ionizing radiation, and affording full anatomic and functional evaluation with CMR together with invasive hemodynamic assessment [9].

We report our experience of CMR fluoroscopy guided RHC in 102 patients, and compare cardiac output and pulmonary vascular resistance measurements using CMR flow versus Fick, under resting conditions and under physiological provocation with inhaled oxygen and nitric oxide.

## Methods

Consecutive patients referred for cardiac catheterization between February 2011 and March 2016 were invited to undergo CMR guided RHC. The research protocol was approved by the Institutional Review Board (NCT01287026). All patients gave written informed consent. Patients were excluded for cardiovascular instability (including ST-elevation myocardial infarction, refractory angina, or refractory congestive heart failure), pregnancy or nursing, an estimated glomerular filtration rate < 30 mL/min/1.73m^2^, or ineligibility for CMR. Baseline demographic and clinical characteristics, invasive hemodynamic and CMR findings, technical details, and procedural complications were prospectively recorded.

### Real-time CMR guided cardiac catheterization

**Fig. 1:**
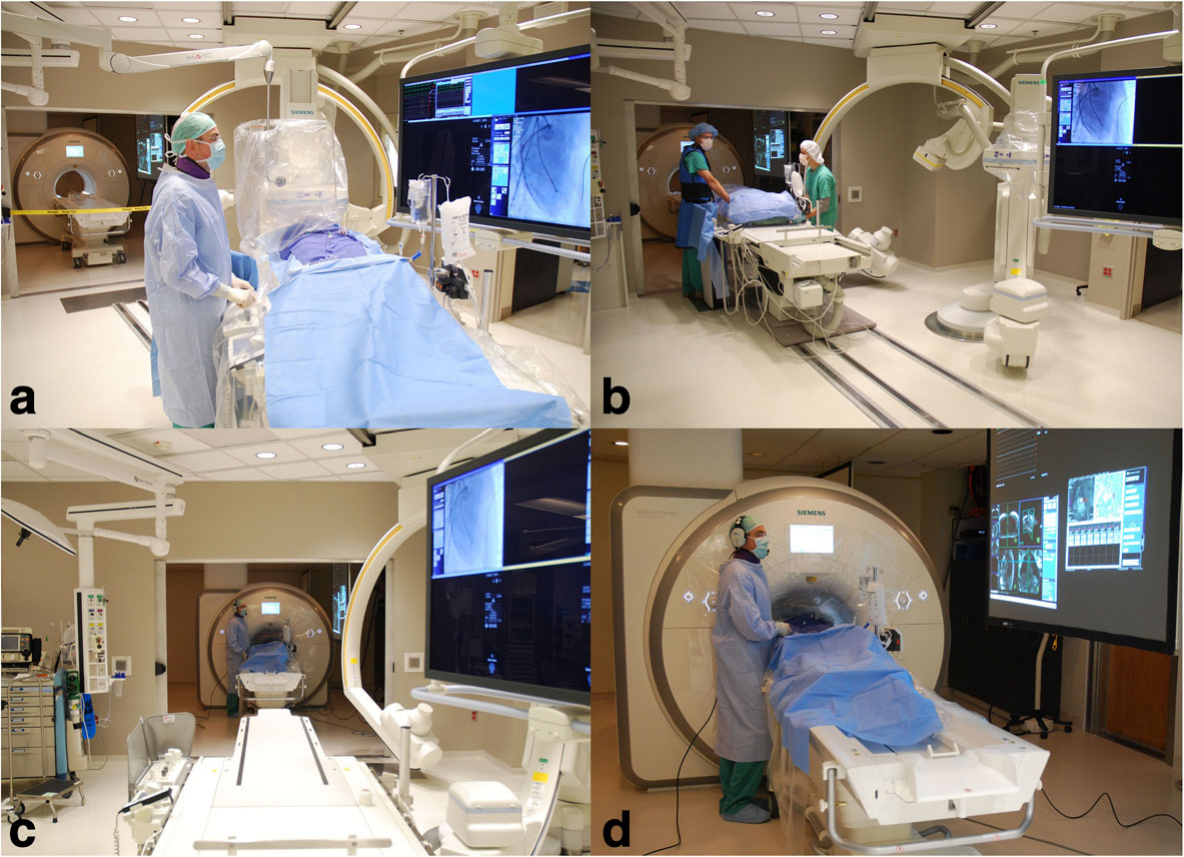
Interventional CMR (iCMR) cardiac catheterization laboratory. **a** Vascular access is obtained in the X-ray room. **b** The patient is transferred from X-ray to CMR using a transfer table that docks with the CMR table. Hemodynamic monitoring is seamless between the two modalities. **c** View of the CMR room from the X-ray side of the iCMR laboratory. **d** Transfemoral cardiac catheterization under CMR guidance. Real-time images are projected in the CMR room for the operator to view. Noise-cancelling headsets permit open communication with the iCMR control room

**Fig. 2:**
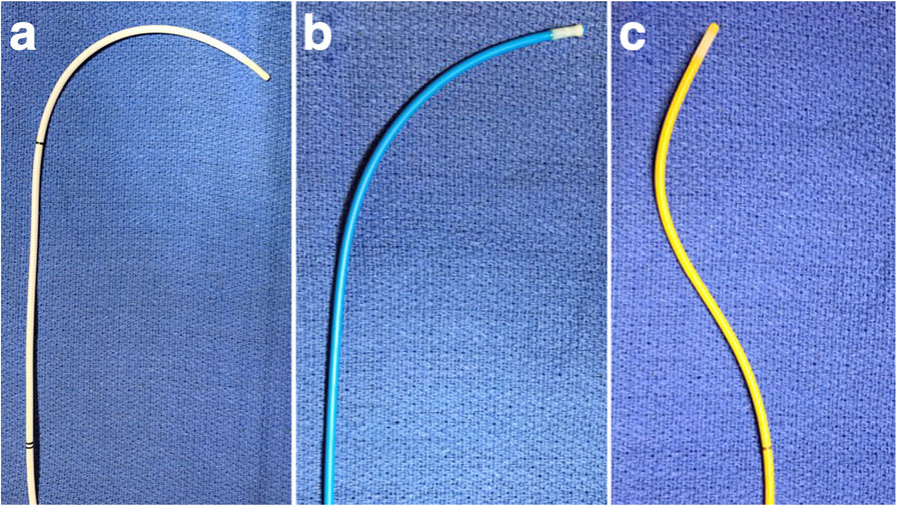
Example CMR-safe catheters for right heart catheterization. A selection of different shape and stiffness CMR-safe balloon catheters is useful to navigate different right heart anatomies. **a** Vascor Balloon Wedge Pressure Catheter, Model #172-110P; (**b**) Medtronic Pulmonary Wedge Pressure Catheter, Model #150075; (**c**) Edwards True Size Monitoring ‘S’ Tip Catheter, Model #S111F7

**Fig. 3:**
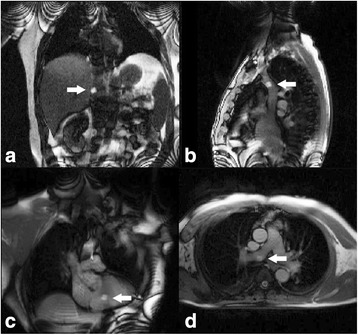
CMR fluoroscopy guided right heart catheterization. **a** Coronal view with the gadolinium filled balloon at the tip of the catheter in the inferior vena cava (arrow), (**b**) sagittal view of the superior vena cava, (**c**) coronal view of the right ventricle, and (**d**) axial view of the main pulmonary artery bifurcation with the balloon in the right pulmonary artery

**Fig. 4:**
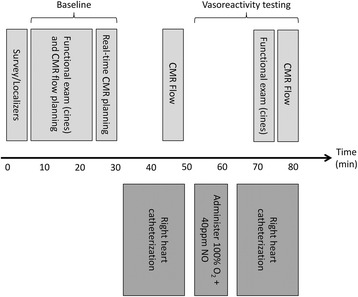
Procedure workflow for CMR guided right heart catheterization

CMR guided RHC procedures were performed under moderate sedation. Moderate sedation is a drug-induced depression of consciousness during which patients respond purposefully to verbal commands, either alone or accompanied by light tactile stimulation, with no interventions required to maintain a patent airway or spontaneous ventilation. All procedures were performed in the catheterization laboratory at the NIH Clinical Center in Bethesda, Maryland (Fig. 1). The laboratory comprises a 1.5 Tesla CMR scanner adjacent to a conventional X-ray fluoroscopy catheterization laboratory, with the ability to easily transfer patients between the two modalities. More details of the physical setup of an CMR catheterization laboratory and technique for CMR fluoroscopy guided RHC have been published previously [8, 10]. At our institution, in addition to the interventional cardiologist operator, a cardiovascular technologist, CMR technologist, cath lab nurse and moderate sedation nurse are present for every procedure. Vascular access was obtained in the adjoining X-ray room using ultrasound guidance. If indicated, left heart catheterization and selective coronary angiography was performed under X-ray guidance. Patients were then transferred to the CMR room, by docking the X-ray and CMR tables (Fig. 1). Seamless hemodynamic monitoring with 12-lead ECG, O_2_ saturation, end-tidal CO_2_ and invasive pressure waveforms was maintained throughout the transfer. Baseline CMR scanning was performed followed by CMR guided RHC using an intrinsically CMR-safe balloon wedge end-hole catheter (Fig. 2), with the balloon filled with dilute gadolinium contrast. The catheter was navigated through the heart under real-time CMR guidance (Fig. 3). Catheterization time was defined as the total time required to navigate catheters through the heart and vasculature, perform pressure measurements, acquire CMR data and sample blood for oxygen saturation (Fig. 4).

Patients underwent vasoreactivity testing if there was a clinical indication, specifically elevated pulmonary artery pressures, using a combination of inhaled nitric oxide (40 ppm) and 100% oxygen. If procedural workflow time permitted, additional patients with normal pulmonary artery pressures also underwent vasoreactivity testing to serve as controls. Pulmonary vascular resistance (PVR) was calculated using the formula PVR = (mean pulmonary artery pressure – mean pulmonary artery wedge pressure)/cardiac output. Cardiac output was obtained with phase contrast CMR and the Fick principle, resulting in two PVR measurements. Indexed PVR (PVRi) was calculated using the formula PVRi = PVR / body surface area. Estimated oxygen consumption (VO_2_) using the LaFarge-Miettinen formula was used for cardiac output calculations with the Fick principle [2]. CMR flow analyses for cardiac output were performed using syngo Argus (Siemens, Erlangen, Germany) or QMass (Medis Medical Imaging Systems, Leiden, Netherlands).

### CMR sequences

Cardiac volumes and function were obtained from real-time balanced steady state free precession (bSFP) cine imaging with repetition time (TR)/echo time (TE) 3.01/1.29 ms; flip angle 62°; bandwidth 558 Hz/pixel; field of view (FOV) 350mmx262mm; matrix 160x92pixels; slice thickness 8 mm; 30 cardiac phases; 11–13 short-axis slices. Images were acquired free-breathing and reconstructed with retrospective gating and respiratory motion correction. Reconstruction was implemented in a distributed cloud computing environment to limit reconstruction latency [11, 12]. To measure cardiac output, phase contrast flow imaging was used with TR/TE 5.0 ms/2.77; flip angle 20°; bandwidth 454 Hz/pixel; FOV 350mmx350mm; matrix 256x256pixels; slice thickness 6 mm; 3 averages; velocity encoding (VENC) set appropriately for individual patient. Surface ECG or invasive arterial blood pressure waveform was used for cardiac gating. RHC was performed with an interactive real-time bSFP sequence with up to 3 interleaved slice planes and with TR/TE 2.6 ms/1.3 ms; flip angle 45°; bandwidth 1008 Hz/pixel; FOV 400mx400mm; matrix 160x120pixels; slice thickness 8 mm. A flow sensitive dark-blood saturation pulse was used for passive visualization of contrast filled balloon. The generalized autocalibrating partially parallel acquisition (GRAPPA) acceleration factor was adjusted interactively by the user between 1 and 4 to balance spatial and temporal resolution as needed for each procedure step.

### Statistical analysis

Data were analyzed with SPSS v19.0. (International Business Machines, Chicago, IL) Categorical variables are presented as percentages and continuous variables as mean ± standard deviation. All tests were two-tailed, and a *p* value of <0.05 was considered significant.

## Results

One hundred ten consecutive patients were invited to participate. Eight patients consented but did not ultimately undergo CMR guided RHC, including 2 patients with claustrophobia, 4 with coronary artery disease and acute chest pain and/or shortness of breath prior to starting the procedure, 1 patient who exceeded the CMR table weight limit and 1 patient with occluded external iliac veins. Therefore, 102 patients underwent CMR guided RHC, including the first 16 patients reported previously [8]. Table 1 summarizes baseline demographic and clinical details of these 102 patients. Ages ranged from 26 to 88 years. Body surface area ranged from 1.2 to 2.4m^2^. RHC was clinically indicated in 75% of patients. The remaining 25% of patients were referred for other procedures such as selective coronary angiography, and consented to undergo additional CMR guided RHC for research purposes only. Of those patients with a clinical indication for RHC, 51% were under investigation for unexplained dyspnea, 25% required investigation of pulmonary hypertension suggested by echocardiography, 21% had a non-invasive diagnosis of congenital heart abnormality (most commonly unrepaired atrial septal defect), and the remaining 3% were referred for other reasons.

**Table 1:** Baseline demographics and clinical details

	All patients (*n* = 102)
Age (years)	53 ± 14
Women	47%
Body surface area (m^2^)	1.8 ± 0.2
Hypertension (%)	53%
Diabetes mellitus (%)	20%
Hyperlipidemia (%)	25%
Tobacco prior or current (%)	30%
Coronary artery disease (%)	37%
Congenital heart disease (%)	16%
Prior cardiac surgery (%)	9%
Valvular heart disease (%)	14%
DVT or PE	14%
Congestive heart failure (%)	16%
Pulmonary arterial hypertension (%)	18%
Pulmonary parenchymal disease (%)	4%
Connective tissue disease (%)	6%
Sickle cell disease (%)	6%

Categorical variables are presented as percentages and continuous variables as mean ± standard deviation. *DVT* deep vein thrombosis; *PE* pulmonary embolism

### Procedural success and timings

Procedural success, defined as successful completion of a full RHC, was met in 95% of patients. In 5 patients, the pulmonary artery could not be reached with a catheter alone. These patients were transferred back to the X-ray room. RHC was successfully completed in all 5 patients under X-ray guidance with the aid of a guidewire. Overall, procedure time was 83 ± 22 min including a combination determined by clinical necessity of left heart catheterization, selective coronary angiography, CMR imaging, baseline RHC and RHC under physiological provocation with inhaled 100% O_2_ and 40 ppm NO. Time to complete a baseline RHC was 20 ± 11 min, and time to complete a second RHC under physiological provocation with inhaled 100% O_2_ and 40 ppm NO was 22 ± 10 min.

### Safety

With moderate sedation, all 102 patients tolerated CMR scanning. No complications related to CMR guided RHC occurred in any patient, and no patient required evacuation from the CMR scanner for hemodynamic instability or resuscitation. Nonetheless, we perform regular emergency evacuation drills and can evacuate a patient from the CMR room to a safe location for defibrillation (the X-ray room in our institution) within less than 1 min. Post-procedure, 7 patients reported access site bruising, of which 2 had palpable hematoma. All were managed conservatively. 1 patient reported generalized urticaria, presumed due to allergy to iodinated contrast administered for selective coronary angiography in X-ray before CMR guided RHC.

### CMR guided RHC hemodynamic findings

Thirty-eight patients (37%) had baseline pulmonary hypertension (mean PA pressure greater than or equal to 25 mmHg). Baseline mean PA pressure was 39 ± 12 mmHg vs. 18 ± 4 mmHg (*p* < 0.001) in patients with and without pulmonary hypertension respectively. Baseline RV end diastolic volume was 104 ± 64 mL vs. 74 ± 24 mL (*p* = 0.02), RV end-systolic volume was 49 ± 30 mL vs. 31 ± 13 mL (*p* = 0.004), RV stroke volume was 55 ± 36% vs. 43 ± 16 mL (*p* = 0.09), and RV ejection fraction was 50 ± 14 vs. 54 ± 17% (*p* = 0.22) in patients with and without pulmonary hypertension respectively.

Baseline cardiac index was 2.8 ± 1.0 L/min/m^2^ vs. 2.9 ± 1.1 L/min/m^2^ (*p* = 0.12) by CMR flow vs. Fick. With inhaled 100% O2 and 40 ppm NO, cardiac index was 2.6 ± 0.7 L/min/m^2^ vs. 2.9 ± 1.0 L/min/m^2^ (*p* = 0.01) by CMR flow vs. Fick. For patients with and without pulmonary hypertension respectively, baseline cardiac index using CMR flow was 3.1 ± 1.3 L/min/m^2^ vs. 2.6 ± 0.8 L/min/m^2^ (*p* = 0.07). This discrepancy was largely the result of higher baseline heart rate (76 ± 16 bpm vs. 65 ± 11 bpm, *p* < 0.001) rather than a difference in stroke volume (42 ± 12 mL vs. 40 ± 42 mL, *p* = 0.79).

Table 2 summarizes cardiac catheterization findings in all patients at baseline and in the fifty eight patients (57%) tested for pulmonary arterial vasoreactivity with inhaled 100% O_2_ and 40 ppm NO. Only 5 patients met current guideline criteria for positive vasoreactivity, defined as a reduction in mean pulmonary artery pressure of at least 10 mmHg and to below 40 mmHg [13]. Median baseline pulmonary artery pressure in these 5 patients was 48 mmHg (41–50 mmHg). Using the earlier definition of ≥20% reduction in pulmonary vascular resistance index, 29% of vasoreactivity studies were positive.

**Table 2:** Cardiac catheterization findings

	Baseline (*n* = 102)	100% O_2_ and 40 ppm NO (*n* = 58)
Heart rate (bpm)	70 ± 14	67 ± 13
RA mean (mmHg)	7 ± 4	7 ± 4
RV pressure systolic/diastolic (mmHg)	45 ± 22/8 ± 5	43 ± 24/7 ± 4
PA pressure systolic/diastolic (mmHg)	43 ± 21/18 ± 9	40 ± 20/17 ± 9
PA mean (mmHg)	26 ± 13	25 ± 10
PA pulse pressure (mmHg)	25 ± 13	23 ± 13
PAWP (mmHg)	13 ± 6.3	13 ± 6
Transpulmonary gradient (mmHg)	13 ± 10	11 ± 10
Cardiac index (L/min/m^2^) by CMR flow	2.9 ± 1.0	2.8 ± 0.9
PVRi (WU.m^2^) by CMR flow	4.5 ± 3.5	4.0 ± 4.7
BP systolic/diastolic (mmHg)	136 ± 22/67 ± 12	140 ± 23/70 ± 11
LVEDP (mmHg)	15 ± 7	n/a
SVC sat (%)	69 ± 8	82 ± 5
IVC sat (%)	70 ± 8	83 ± 4
Mixed venous sat (%)	69 ± 7	83 ± 4
RA sat (%)	71 ± 7	84 ± 9
RV sat (%)	71 ± 7	85 ± 7
PA sat (%)	71 ± 7	84 ± 6
Aorta sat (%)	94 ± 4	98 ± 1

Categorical variables are presented as percentages and continuous variables as mean ± standard deviation. *BP* blood pressure, *IVC* inferior vena cava, *LVEDP* left ventricular end-diastolic pressure, *PA* pulmonary artery, *PAWP* pulmonary artery wedge pressure, *PVRi* pulmonary vascular resistance index, *RA* right atrium, *RV* right ventricle, *SVC* superior vena cava

**Fig. 5:**
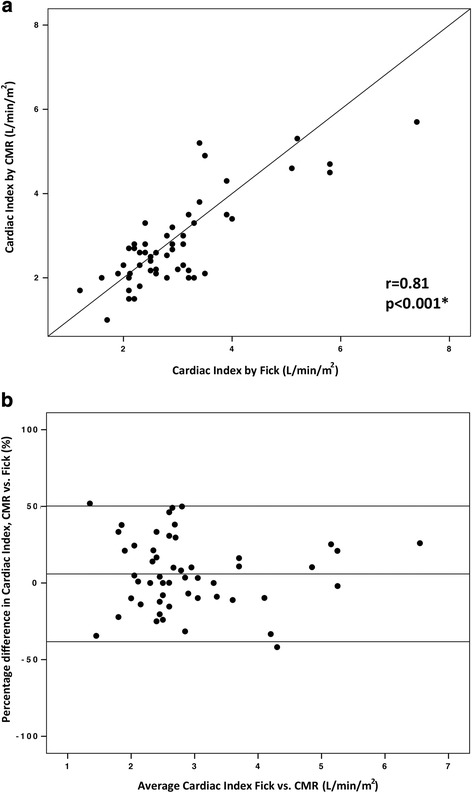
Comparison of cardiac index calculated with the Fick principle and with CMR flow at baseline on room air. **a** Correlation between cardiac index calculated with the Fick principle and with CMR flow at baseline on room air (*n* = 56 paired calculations). **b** Bland-Altman plot of the difference in cardiac index calculated with the two methods, and mean cardiac index with the two methods

**Fig. 6:**
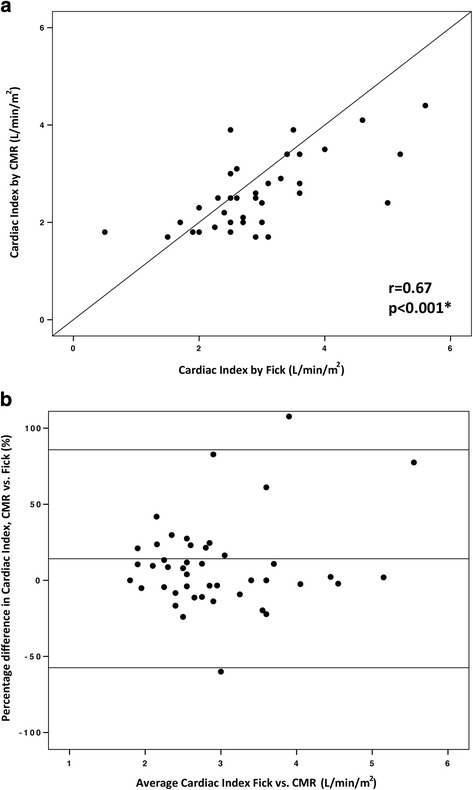
Comparison of cardiac index calculated with the Fick principle and with CMR flow on inhaled 100% O2 and 40 ppm NO. **a** Correlation between cardiac index calculated with the Fick principle and with CMR flow on inhaled 100% O_2_ and 40 ppm NO (*n* = 47 paired calculations). **b** Bland-Altman plot of the difference in cardiac index calculated with the two methods, and mean cardiac index with the two methods

A total of 103 paired cardiac index calculations were available for both CMR flow and Fick principle (56 at baseline on room air, and 47 on inhaled 100% O_2_ and 40 ppm NO). At baseline, the mean difference (or bias) between methods was 5.9% with limits of agreement −38.3% and 50.2%. The correlation coefficient between the two methods was 0.81 (*p* < 0.001) (Fig. 5). Under physiological provocation with inhaled 100% O_2_ and 40 ppm NO, the bias was 14.2% with limits of agreement −57.4% and 85.8%. The correlation coefficient between the two methods was 0.67 (*p* < 0.001) (Fig. 6).

**Fig. 7:**
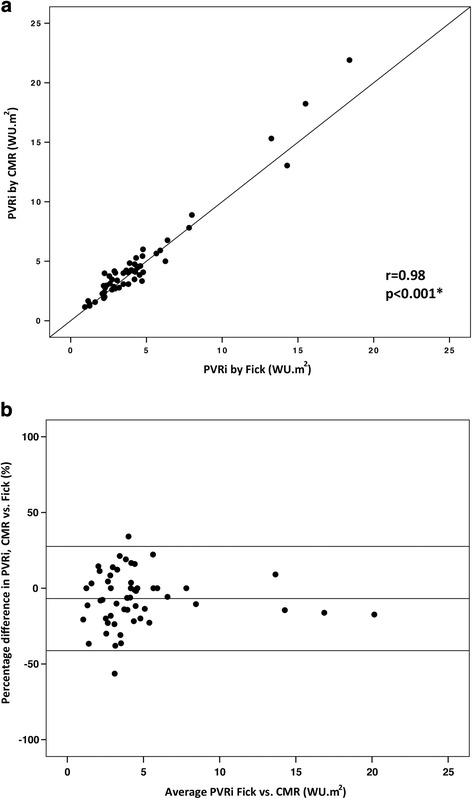
Comparison of PVRi calculated with the Fick principle and with CMR flow at baseline on room air. **a** Correlation between PVRi calculated with the Fick principle and with CMR flow at baseline on room air (*n* = 54 paired calculations). **b** Bland-Altman plot of the difference in PVRi calculated with the two methods, and mean PVRi with the two methods. PVRi: pulmonary vascular resistance index

**Fig. 8:**
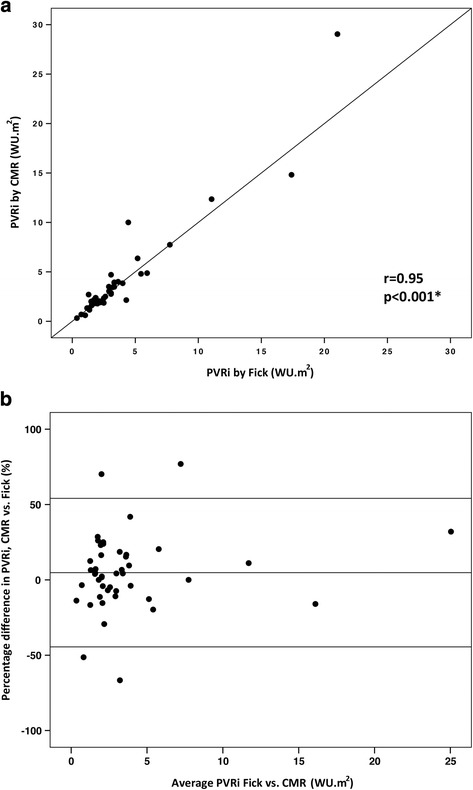
Comparison of PVRi calculated with the Fick principle and with CMR flow on inhaled 100% O2 and 40 ppm NO. **a** Correlation between PVRi calculated with the Fick principle and with CMR flow on inhaled 100% O_2_ and 40 ppm NO (*n* = 45 paired calculations). **b** Bland-Altman plot of the difference in PVRi calculated with the two methods, and mean PVRi with the two methods. PVRi: pulmonary vascular resistance index

A total of 99 paired PVRi calculations (invasive pressure and cardiac output with CMR flow or Fick principle) were obtained. Baseline PVRi was 4.5 ± 3.5 WU.m^2^ vs. 4.8 ± 3.9 WU.m^2^ (*p* = 0.01) by CMR flow vs. Fick. With inhaled 100% O2 and 40 ppm NO, PVRi was 3.9 ± 4.7 WU.m^2^ vs 3.5 ± 3.9 WU.m^2^ (*p* = 0.15) by CMR flow vs. Fick. Across both conditions, the bias between indexed PVR by CMR and Fick was −0.02 WU.m^2^ with limits of agreement −2.6 and 2.5 WU.m^2^. At baseline, mean PVRi was 4.5 ± 3.5WU.m^2^ by CMR flow and 4.8 ± 4.5 WU.m^2^ by Fick principle. The correlation coefficient between the two methods was 0.98 (*p* < 0.001) (Fig. 7). Bland-Altman analysis revealed a bias of −6.8% with limits of agreement −41.2% and 27.6%. Under physiological provocation with inhaled 100% O_2_ and 40 ppm NO, mean PVRi was 4.0 ± 4.7 WU.m^2^ by CMR flow and 3.9 ± 4.1 WU.m^2^ by Fick principle. The correlation coefficient between the two methods was 0.95 (*p* < 0.001) (Figure 8). Bland-Altman analysis revealed a bias of 4.8% with limits of agreement −44.5% and 54.1%.

Table 3 summarizes hemodynamic and CMR findings in response to inhaled 100% O_2_ and 40 ppm NO in patients with or without pulmonary hypertension defined by baseline mean pulmonary artery pressure. Table 4 summarizes hemodynamic and CMR findings in response to inhaled 100% O_2_ and 40 ppm NO according to whether patients were vasoactive or not (defined as ≥20% reduction in PVRi). Analysis per current guideline definition of vasoreactivity (defined as reduction in mean pulmonary artery pressure of at least 10 mmHg and to below 40 mmHg) was not possible due to the low number of patients with positive response (*n* = 5).

**Table 3:** Hemodynamic and CMR findings in response to inhaled 100% O2 and 40 ppm NO in patients with and without pulmonary hypertension

	Baseline	100% O_2_ + 40 ppm NO	*p* value
*Normal pulmonary pressures (n = 30)*			
Mean PA pressure (mmHg)	18 ± 4	16 ± 5	0.01*
Mean PAWP (mmHg)	9 ± 3	10 ± 3	0.008*
Cardiac index (L/min/m^2^) by CMR flow	2.6 ± 0.1	2.5 ± 0.1	0.65
RV end diastolic volume (mL)	74 ± 24	73 ± 24	0.41
RV end systolic volume (mL)	27 ± 12	25 ± 13	0.22
RV stroke volume (mL)	47 ± 15	47 ± 15	0.61
RV ejection fraction (%)	64 ± 9	66 ± 11	0.03*
LV end diastolic volume (mL)	75 ± 20	76 ± 20	0.11
LV end systolic volume (mL)	32 ± 18	33 ± 18	0.10
LV stroke volume (mL)	43 ± 9	43 ± 9	0.93
LV ejection fraction (%)	60 ± 11	59 ± 11	0.18
*Pulmonary hypertension (n = 28)*			
Mean PA pressure (mmHg)	39 ± 12	34 ± 12	<0.001*
Mean PAWP (mmHg)	16 ± 6	16 ± 8	0.75
Cardiac index (L/min/m^2^) by CMR flow	3.1 ± 0.3	3.0 ± 0.2	0.44
RV end diastolic volume (mL)	120 ± 73	112 ± 74	0.01*
RV end systolic volume (mL)	57 ± 35	52 ± 40	0.06
RV stroke volume (mL)	64 ± 41	61 ± 37	0.22
RV ejection fraction (%)	54 ± 12	56 ± 11	0.17
LV end diastolic volume (mL)	89 ± 33	92 ± 34	0.045*
LV end systolic volume (mL)	39 ± 21	42 ± 25	0.07
LV stroke volume (mL)	50 ± 14	50 ± 10	0.94
LV ejection fraction (%)	59 ± 10	58 ± 9	0.22

Categorical variables are presented as percentages and continuous variables as mean ± standard deviation. Cardiac index was derived from CMR flow. *denotes statistical significance. *PA* pulmonary artery, *PAWP* pulmonary artery wedge pressure; *PVRi* pulmonary vascular resistance index, *RV* right ventricle

**Table 4:** Hemodynamic and CMR findings in response to inhaled 100% O2 and 40 ppm NO in patients with and without a 20% reduction in PVRi

	Baseline	100% O_2_ + 40 ppm NO	*p* value
*Positive vasodilatory response (n = 29)*			
Mean PA pressure (mmHg)	26 ± 12	22 ± 10	<0.001*
Mean PAWP (mmHg)	13 ± 6	14 ± 7	0.11
Cardiac index (L/min/m^2^) by CMR flow	3.0 ± 0.2	2.9 ± 0.2	0.06
PVRi (WU.m^2^) by CMR flow	4.2 ± 3.2	2.6 ± 2.2	<0.001*
RV end diastolic volume (mL)	96 ± 63	91 ± 63	0.014*
RV end systolic volume (mL)	39 ± 32	36 ± 35	0.12
RV stroke volume (mL)	57 ± 34	55 ± 32	0.14
RV ejection fraction (%)	62 ± 11	63 ± 12	0.19
*Negative vasodilatory response (n = 15)*			
Mean PA pressure (mmHg)	25 ± 12	24 ± 11	0.08
Mean PAWP (mmHg)	11 ± 5	11 ± 4	0.65
Cardiac index (L/min/m^2^) by CMR flow	2.7 ± 0.8	2.7 ± 0.8	0.98
PVRi (WU.m^2^) by CMR flow	4.9 ± 3.8	4.9 ± 3.4	0.97
RV end diastolic volume (mL)	79 ± 23	81 ± 26	0.44
RV end systolic volume (mL)	35 ± 15	33 ± 17	0.33
RV stroke volume (mL)	45 ± 14	48 ± 12	0.014*
RV ejection fraction (%)	58 ± 11	62 ± 13	0.008*

Categorical variables are presented as percentages and continuous variables as mean ± standard deviation. Cardiac index and PVRi were derived from CMR flow. *PA* pulmonary artery, *PAWP* pulmonary artery wedge pressure, *PVRi* pulmonary vascular resistance index, *RV* right ventricle

## Discussion

CMR fluoroscopy (also known as real-time CMR) allowed catheter navigation for complete RHC in 97/102 patients with a range of cardiovascular diagnoses. The procedure was safe, had a high success rate, and had acceptable catheterization times. This is the largest published series of paired comparisons of cardiac output and PVRi measured by both CMR flow and Fick techniques. We demonstrated excellent agreement between the two methods at baseline and reasonable agreement during physiological provocation with inhaled 100% oxygen and 40 ppm NO, which is a circumstance when Fick is less accurate because of reduced signal-to-noise due to narrow arteriovenous oxygen difference.

We previously tested feasibility of CMR guided RHC, comparing timings of specific procedural steps with X-ray guided RHC [8]. In that experiment, total RHC times (‘sheath to sheath’) were equivalent irrespective of image guidance modality (X-ray 19.4 + 11.5 min, CMR with air-filled balloon 21.4 + 6.0 min, CMR with gadolinium-filled balloon 21.0 + 8.8 min, *p* = 0.347). We also compared relative conspicuity of air-filled versus gadolinium-filled catheter balloons, with a clear operator preference for gadolinium. Integration of dedicated pulse sequences that darken blood while preserving background tissue signal further facilitates catheter visualization. Only the balloon at the tip of the catheter can be visualized, not the catheter shaft, and so the operator must learn to interpret catheter configuration based on balloon tip movement. This is not difficult for an experienced interventional cardiologist to learn. CMR guided RHC has been designated a standard clinical procedure at NIH. All patients are now offered CMR guided catheterization, even if they are not participating in research.

Vascular access was obtained in the adjoining X-ray room using ultrasound guidance, as there are currently no commercially available CMR-conditional guidewires to enable access to be obtained close to the CMR scanner. Fluoroscopy is certainly not required and therefore any adjoining room could be used for vascular access. An adjoining X-ray room does allow selective coronary angiography to be performed in the same sitting as CMR guided RHC. If needed, a patient can also be transferred from CMR to X-ray for a planned or bailout intervention. If general anesthesia is used, as is common in pediatrics, both CMR and cardiac catheterization can be performed during a single general anesthesia, sparing the child the need for a second.

We have learned that in the absence of available CMR-conditional guidewires, it is prudent to have several differently shaped balloon catheters to maximize procedural success (Fig. 2). Depending on operator choice of central venous approach and patient anatomy, straight or angled catheters are more appropriate to reach target chambers or vessels. We failed to reach the pulmonary artery in 5 patients with severely dilated right heart chambers caused by pulmonary hypertension. In all 5 patients, pulmonary artery catheterization under X-ray guidance was also unsuccessful without the aid of a guidewire. CMR-conditional guidewires are in development, which should improve the procedural success rate [14, 15]. CMR-conditional guidewires will also permit more complex interventions to be performed under CMR guidance.

Mean PVRi at baseline was 5.1 ± 6.3WU.m^2^ in the original report by Muthurangu et al. in which PVRi comparisons using the Fick principle and CMR flow were made in 15 patients [6]. The correlation coefficient at baseline on room air was excellent in their study (*r* = 0.91, *p* < 0.05). The bias between the two methods was 2.3% with limits of agreement −45.5% and 50.2%. On inhaled 100% O_2_ and 20 ppm NO, the correlation coefficient was poor (*r* = 0.59, *p* = 0.02). Bias between the two methods was 54.2% with limits of agreement −66.0% and 174.4%. Our results, with substantially larger number of paired calculations, also showed a strong correlation between the two methods at baseline on room air. We observed a stronger correlation on inhaled 100% O_2_ and 40 ppm than that reported by Muthurangu et al., although the limits of agreement were still much wider than at baseline. This may be due to a number of factors, including higher number of paired calculations in our study, use of estimated rather than measured VO_2_, and lower mean PVRi at baseline.

We tested the hypothesis that current criteria for positive vasoreactivity to inhaled 100% O_2_ and 40 ppm NO, defined as ≥10 mmHg reduction in mean pulmonary artery pressure and to below 40 mmHg, may miss subtle RV changes to pulmonary vasodilation. Using the older and less stringent criterion for positive vasoreactivity of ≥20% reduction in PVRi, we did observe small differences in RV volumes and ejection fraction between groups (Table 4). Although some of these differences met statistical significance, they were not clinically meaningful. More information is needed to address whether evaluating RV response to pulmonary arterial vasodilators has prognostic or therapeutic merit. Even without vasodilator testing, patients in this study with pulmonary hypertension had increased RV end-diastolic and end-systolic volumes, and a trend towards lower stroke volumes and ejection fraction at baseline. For these patients, this incremental anatomic and functional information proved useful to guide treatment and inform prognosis because RV function is one of the strongest prognostic factors in patients with pulmonary hypertension.

### Future directions

We believe that exercise CMR catheterization is a very promising clinical tool to unmask latent pathology, particularly in patients with unexplained dyspnea and normal non-invasive and invasive testing at rest. Most cardiac symptoms are not apparent at rest, and only manifest with stress. Exercise is unquestionably the most physiological form of stress. Barber et al. elegantly demonstrated the feasibility and value of cardiopulmonary exercise testing in a CMR scanner [16]. Quantification of RV dimensions and function during exercise is feasible using real-time CMR sequences [17]. We previously validated free-breathing motion-corrected cine imaging for cardiac chamber volume and function analysis [18]. Other groups have shown that exercise during CMR catheterization is feasible [19, 20] and CMR ergometers that permit carefully titrated workload are already commercially available.

### Limitations

Due to protocol-specified procedure time limitations, we did not perform repeated measurements of cardiac output using CMR flow and Fick principle. Therefore, it is not possible to comment on repeatability of either technique. Neither did we perform an analysis of intra- or inter-observer variability.

CMR guided cardiac catheterization requires additional infrastructure. Most important, noise-suppressing headsets for communication, screens to visualize real-time images for the operator in the CMR room, and hemodynamic recording system. Many of these elements are now commercially available. It is possible to equip existing diagnostic CMR rooms to perform CMR guided invasive cardiac catheterization. The major limitation to performing more complex CMR guided interventions remains lack of CMR-conditional devices, catheters and guidewires. However, key components are now commercially available or under development, including electrophysiology ablation and mapping system [21], CMR endomyocardial bioptome [22], and CMR-conditional guidewires.

## Conclusion

In conclusion, real-time CMR fluoroscopy guided RHC is feasible with high procedural success rate, acceptable procedure times and excellent safety. Cardiac output and pulmonary vascular resistance quantification with CMR flow correlated well with Fick principle at baseline, and under physiological provocation with inhaled 100% oxygen and NO. Using this approach, it is possible to perform high quality invasive hemodynamic studies together with anatomic and functional assessment in patients with cardiopulmonary disease using solely CMR guidance.

## Abbreviations

CMR: Cardiovascular magnetic resonance
GRAPPA: Generalized autocalibrating partially parallel acquisition
LV: Left ventricle
PVR: Pulmonary vascular resistance
RHC: Right heart catheterization
RV: Right ventricle
